# A Time Scheduling Model of Logistics Service Supply Chain Based on the Customer Order Decoupling Point: A Perspective from the Constant Service Operation Time

**DOI:** 10.1155/2014/756178

**Published:** 2014-03-12

**Authors:** Weihua Liu, Yi Yang, Haitao Xu, Xiaoyan Liu, Yijia Wang, Zhicheng Liang

**Affiliations:** College of Management and Economics, Tianjin University, Tianjin 300072, China

## Abstract

In mass customization logistics service, reasonable scheduling of the logistics service supply chain (LSSC), especially time scheduling, is benefit to increase its competitiveness. Therefore, the effect of a customer order decoupling point (CODP) on the time scheduling performance should be considered. To minimize the total order operation cost of the LSSC, minimize the difference between the expected and actual time of completing the service orders, and maximize the satisfaction of functional logistics service providers, this study establishes an LSSC time scheduling model based on the CODP. Matlab 7.8 software is used in the numerical analysis for a specific example. Results show that the order completion time of the LSSC can be delayed or be ahead of schedule but cannot be infinitely advanced or infinitely delayed. Obtaining the optimal comprehensive performance can be effective if the expected order completion time is appropriately delayed. The increase in supply chain comprehensive performance caused by the increase in the relationship coefficient of logistics service integrator (LSI) is limited. The relative concern degree of LSI on cost and service delivery punctuality leads to not only changes in CODP but also to those in the scheduling performance of the LSSC.

## 1. Introduction

At present, with the growing demand for customized logistics services, many logistics enterprises not only provide customers with mass service but also meet the demand for customized service as well as consider changes in the logistics service mode. Specially, these enterprises attempt to provide mass customization logistics services (MCLS) instead of mass logistics services [1]. In the MCLS environment, to meet the individualized service requirements of customers and achieve the necessary capabilities for offering mass service, many logistics enterprises form a logistics service supply chain (LSSC) by means of unions and integration [2, 3]. In MCLS, the key factor in realizing the competitiveness of the LSSC is whether it can offer customized service with the cost of mass service through reasonable scheduling.

The main participants of MCLS are customers, functional logistics service providers (FLSPs), and logistics service integrators (LSIs). The LSI, as the core enterprise of the LSSC, needs to handle the logistics services demand of multiple costumers, analyze different service processes required by each customer, and integrate these similar orders to achieve mass service effects. As different FLSPs have advantages both in different logistics service processes and different logistics service functions, LSIs need to integrate the different advantages of FLSPs to provide customers with customized logistics service. Thus, the effect of a customer order decoupling point (CODP) location on scheduling performance must be taken into consideration. Customer order decoupling point (CODP) is a concept frequently used in distinguishing Make-to-Stock (MTS) operations from Make-to-Order (MTO) operations [4]. The concept of CODP is widely used in the production and manufacturing fields and has become the main content of researches on postponement strategies.

The CODP is the demarcation point of mass service and customization service both in product supply chain field and service supply chain field, but significant differences exist in the CODPs of these two fields. On the one hand, in the product supply chain, CODP is always regarded as the inventory holding point of the component commonality in order to meet customization demand from downstream customers quickly. In contrast, in the service supply chain there is no inventory as service cannot be stored. On the other hand, in the manufacturing supply chain, the component commonality before CODP usually could be produced ahead of time and held as work-in-process (WIP). On the contrary, in the service supply chain, service cannot be mass-produced ahead of schedule since service has a characteristic of inseparability [5]; that is, for service the production and consumption are usually going on at the same time. Thus, in service supply chain, either the process before the CODP or the one after the CODP depends on a specific customer order. Given the reasons above, the scheduling problem of the service supply chain confronts more dynamics and uncertainty when compared to the one of the manufacturing supply chain.

Completion time is an important index for logistics service level. The time scheduling problem is one of key problems in logistics service scheduling. Numerous logistics companies have paid much attention to improve the time scheduling performance while offering mass customization logistics service to customers. For example, the e-commerce transactions of China Taobao online mall amounted to 3.36 billion in November 11, 2011. In only one day, Yuantong Express Delivery received from all its branch companies in China up to 2.67 million parcels, which is more than four times the number in the same period in 2010. Express parcels must be delivered in a customized method within three to five days to customers, which are located in 31 provinces in China. This requirement caused tremendous pressure on Yuantong Express Delivery. Meeting the individual logistics service requirements within a certain period of time became a great problem for Yuantong Express Delivery. They needed to combine similar orders and set a CODP for all orders while considering the time requirement of costumers. Ahead of the CODP, Yuantong Express Delivery carried out mass service catering to all orders and then offered customized service to each order. Apparently, the influence of CODP location on the LSSC time scheduling performance should be considered. Another example is as follows: there are three customers in Tianjin city in request of delivery service to three cities in Eastern China, respectively, which are Hangzhou, Ningbo, and Jinhua city. The required delivery completion time is 48 hours for the order to Hangzhou, 60 hours for that of Ningbo, and 72 hours for that of Jinhua. Obviously, it is not economical to deliver these three orders, respectively, using different trucks. Thus, Yuantong Express Delivery always first consolidates the three orders and sends them to a place (such as Shanghai) from Tianjin in a time, which is mass delivery service. After arriving in Shanghai, customization service is conducted. Yuantong Express Delivery will assign different trucks to deliver the three orders' parcels, respectively, to Hangzhou, Ningbo, and Jinhua. Therefore, Shanghai becomes the CODP of the whole logistics process. In practice, the selectable CODP is not confined to Shanghai and other cities, such as Nanjing and Suzhou, may be alternative options. Various decisions of the CODP would lead to a variety of scheduling results. As this example indicates, the CODP is the demarcation point of mass service and customization service, and it directly determines the service process sequence, the service time, and the service cost. Thus, the impact of the CODP decision on the LSSC scheduling performance must be taken into consideration.

In our previous study [6], we have come up with the basic model of time scheduling in the LSSC and explored the delay coefficient of customer order completion time and relationship cost coefficient of the LSI on the time scheduling result in MC environment. In addition, we have compared the effects of two behaviors, decreasing the delay coefficient of customer order completion time and relationship cost coefficient of the LSI, which both can improve the supply chain performance to some extent. But what Liu et al. [6] considered was the operation situation after the CODP position has been decided which means that the CODP position is a known parameter and the CODP decision problem has not been taken into consideration. In actual scheduling operation, the time constraints will affect the CODP position option. Furthermore, the CODP position will have a great influence on the time scheduling performance. Thus, it is necessary to consider the influence of the CODP on time scheduling results. This paper delves into constructing the LSSC time scheduling model in consideration of the CODP decision problem.

The CODP positioning problem and the time scheduling problem have caught the attention of many researchers. Existing research on the supply chain scheduling model has four deficiencies, which will be discussed in this paper.

First, the conclusions drawn from traditional manufacturing supply chain scheduling models may not be fully applicable to LSSC scheduling problems and it is necessary to consider the characteristics of the service supply chain in the new model. On the one hand, the CODP model of the service supply chain does not include inventory cost since service production is different from manufacturing production. But in the situation of multiorders, a service company also faces the service order pile-up problem in the transition session from the mass service process to the customization service process. Then, how to introduce this cost factor in the objective function? On the other hand, under circumstances of multiorders, the lead times of different service orders are different [7], so are the service processes. Then how to reflect these lead-time constraints and the differences of service processes in the model constraints?

Second, as an important index reflecting supply chain agility, the time requirement of costumers may change in a number of cases (see, e.g., [8, 9]). The operation time requirements to FLSPs may have some variation [7]. Thus, the influence of service completion time on costumers (when service time is ahead of schedule or delayed) or FLSPs on the scheduling results should be considered. When a service is ahead of schedule or delayed, how then is completion time expressed in the model? If the variation of completion time is ahead of time or delayed, what is its effect on supply chain performance?

Third, most of the existing time scheduling models of the supply chain concern only the cost control goal and the time requirement constraint. However, for the LSSC under the MCLS environment, cost control is not always the most important scheduling goal. The punctual delivery of service orders and satisfaction of FLSPs are also of great importance. Moreover, CODP positioning does not only influence the service operation cost but also the time scheduling effects. Therefore, how to present these objective functions in the model and how can the CODP constraint be reasonably expressed?

Fourth, it is necessary for the LSI managers to know which parameters have an influence on scheduling results. Then in this paper, what scheduling parameters will affect scheduling results? What are the specific influence rules? For the LSI, how to better deal with the time scheduling problem using these influence rules?

The LSSC time scheduling model proposed in this paper solves these four problems mentioned above. To minimize the total order operation costs of the LSSC, minimize the difference between the expected and actual time of completing the service orders, and maximize the satisfaction of functional logistics service providers, this study establishes an LSSC time scheduling model considering the CODP. The results show that the conclusion obtained from our previous study could be extended when considering the CODP decision problem. First, the optimal scheduling table is flexible to some degree; that is, the order completion time of the LSSC is allowed for ahead-of-time and delay within a certain range. As the requests for completing orders in advance become more intensive, the number of optional CODP positions decreases, which leads to either the decrease of the LSSC flexibility or the customization degree of customer orders. Second, the improvement of the supply chain comprehensive performance caused by the increase of the relationship cost coefficient of the logistics service integrator (LSI) is limited. Furthermore, the difference of the LSI's preference for cost and service delivery punctuality leads not only to the difference of the CODP position but also the scheduling performance of the LSSC.

This paper is organized as follows.  Section 2 presents the literature review, in which the existing supply chain optimization scheduling models and methods as well as the CODP positioning method are systematically summarized. In Section 3, an LSSC time scheduling model based on the CODP is established.  Section 4 presents the model solution, in which a method for solving the multi-objective programming model is provided.  Section 5 gives the numerical analysis, in which the influence of relevant parameters on the time scheduling performance is explored. The main conclusions and management insights are discussed in Section 6. The reference value for researchers and managers are given respectively. The last section presents the limitation of this study and provides further research directions in this field.

## 2. Literature Review

The literature review presents related studies on the time scheduling of the LSSC and the CODP positioning problem under the MCLC environment. Our research direction is proposed after we summarize research development and its deficiencies.

### 2.1. Mass Customization (MC), CODP, and Postponement Strategy

Since Pine and Davis [10] proposed that the MC mode would become the new frontier in business competition in 1993, after nearly 20 years of development and application, the MC mode has increasingly become a mainstream mode of operation. According to da Silveira et al. [11], MC is the ability to provide customized products or services through flexible processes in high volumes and at reasonably low costs. MC has been extensively studied and applied in the field of manufacturing supply chains because of its significant improvement in operation performance. Many scholars have conducted studies on this topic. Fogliatto et al. [12] conducted a detailed review of the literature on MC production since the 1980s. From the view of the current progress in domestic and international research, research on MC mainly developed in the MC production mode in the manufacturing industry, such as the MC mode and its product development (see, e.g., [13, 14]), production planning and control technology in MC (see, e.g., [15, 16]), costs of MC (see, e.g., [17]), and factors and conditions that influence MC ( see, e.g., [18]), among others.

CODP is an important topic in the study of MC production. Yang et al. [19] point out that CODP, as the core element in realizing the MC mode, is one of the effective methods for managing uncertainty. The reasonable positioning of the CODP largely determines the cost and degree of the customization of a supply chain. Therefore, a number of researchers have conducted in-depth studies on the CODP (see, e.g., [20]), from single CODP positioning to multi-CODP positioning (see, e.g., [21, 22]) and from static research to dynamic research (see, e.g., [23]).

Postponement is closely related to CODP. The concept “postponement” was first introduced by Alderson [24] in his paper* Marketing efficiency and the principle of postponement.* He defined postponement as a marketing strategy of putting off the changes in forms and features as much as possible. Postponement manufacturing strategy has been widely used in supply chain researches. Shapiroe [25] studied the postponement strategy positioning problem and established the correlation between the supply chain and the postponement strategy. According to the difference in the degree of customization requested by customers, Bowerson and Closs [26] divided the postponement strategies into three categories, which are Time Postponement, Place Postponement, and Form Postponement. Larry et al. [27] developed a new methodology for analyzing the impact of forecast accuracy on the decision to postpone production. In the implement of postponement manufacturing, the CODP position is critical. By changing the CODP position, postponement degree is increased or decreased, and then accordingly the degree of customization is adjusted. On the one hand, enabling manufacturing postponement can provide firms with a prompt response [28]. On the other hand, postponement has recently been mentioned as a useful tool for managing supply risk and disruptions [29].

In recent years, studies on CODP in the service industry have become a research focus. Since the CODPs in service industry and manufacturing industry share similarities and show differences (as mentioned in Section 1), some researchers have already applied CODP theory from the manufacturing industry to the service industry. For example, Tang and Chen [30] applied postponement theory originated from manufacturing to operational management in the service industry and discussed the factors that needed to be considered in CODP positioning. However, the study was limited to a single service enterprise. Specialized research on the CODP for a general service supply chain, especially for LSSC, is lacking. Therefore, considering the features of the service supply chain, discussing the CODP positioning problem in the service supply chain, and analyzing the effect of CODP on supply chain scheduling performance are necessary.

### 2.2. Time Scheduling of Supply Chain

Most previous research on supply chain scheduling focused on the manufacturing industry and achieved substantial results. In 2003, Hall and Potts [31] published “*Supply chain scheduling: batching and delivery*,” an early systematically research on the supply chain scheduling model. Many earlier studies on supply chain scheduling paid attention to the job shop scheduling within a single enterprise, such as Lee et al. [32] and Philipoom [33]. The main concern of these studies is the arrangement of processing procedures and the order operation sequence. Some scholars studied assembly system coordination in manufacturing enterprises (see, e.g., [34]). Studies on supply chain scheduling in the MC production mode have emerged in recent years (see, e.g., [35, 36]).

In terms of supply chain time scheduling, some scholars conducted dominant contradiction analysis and studied supply chain scheduling optimization solutions in MC (see, e.g., [37]). Apparently, some differences exist between the ideal scheduling timetables of different supply chain members and those of the customer demand. Dawande et al. [38] explored ways to solve this discrepancy, consequently inspiring us to conduct our research. Based on the integration of supply chain production planning and the scheduling process, Mishra et al. [39] designed a mixed integer programming model. Similar to the CODP positioning problem, cost is the primary factor considered in supply chain scheduling (see, e.g., [40, 41]). Most studies assume that the order completion time required by customers or the delivery time required by suppliers is fixed. However, as important index reflecting supply chain agility, the time requirements of customers may change in a number of cases (see, e.g., [8, 9]). Moreover, the operation time requirements to FLSPs do not have strict limitations, enabling a certain amount of variation [7]. The influence of service completion time, whether ahead of schedule or delayed by customers or FLSPs, on the scheduling results should be considered. Aside from the cost objective, the punctual delivery of service orders and the satisfaction of FLSPs directly influence customer satisfaction as well. Therefore, considering the influence of the differences among the degrees of importance of the objective functions on supply chain performance is necessary. However, the current literature has not addressed this issue.

Although research on supply chain scheduling in the MC environment has increasingly improved, studies on the service supply chain remain significantly insufficient. Similar to that on the manufacturing supply chain, research on the service supply chain mainly focuses on service process scheduling (see, e.g., [30]) and order assignment scheduling (see, e.g., [4, 42]), among others. However, only a few studies have been made on time scheduling, which is an important part of LSSC scheduling. Carrying out research on time scheduling combined with a service supply chain (especially LSSC) in certain industries is necessary.

From the review of relevant literature, it can be concluded that specialized research on CODP for service supply chain, especially for LSSC, is still insufficient as well as research on the time scheduling of LSSC. Under the MCLS mode, the time scheduling of LSSC should consider the influence of CODP on scheduling results. Furthermore, we find that three important issues remain unresolved. First, under the MCLS environment, how to introduce the mass characteristics and the personalized characteristics of the customization service? Second, in the case of multiservice orders, the lead-times of different service orders are different [7], so are the service processes. How to express the lead time constraints of customer orders? How to demonstrate the difference of the service process required by customer orders in the constraints? Third, the scheduling model always has multiple objective functions in which time objective (the punctual service delivery objective) and the cost objective coexist. The relative concern degree of LSI on cost and service delivery punctuality may change in different environments. Thus, what is the effect of these changes on scheduling performance? These three problems are discussed in the Model Building Section of this paper.

## 3. Model Building

In this section, the LSSC time scheduling model considering CODP in the case of multiservice orders is established. This scheduling model is a multiobjective programming model since in the scheduling process, the LSI needs to consider three scheduling objectives, which are minimizing the total order operation costs of the LSSC, minimizing the difference between the expected and actual time of completing the service orders, and maximizing the satisfaction of functional logistics service providers. In addition, some constraints need to be met in this scheduling model, including the completion time constraints required by customers, the time correlation constraint between the upstream process and the downstream one, and the satisfaction constraint.

In Section 3.1, we describe the problem and the sequence of events involved in the model. In Section 3.2, the important model assumptions are proposed. In Section 3.3, the LSSC time scheduling model, which is a multiobjective programming model, is presented.

### 3.1. Problem Description

A two-echelon LSSC composed of one LSI and many FLSPs are assumed. The LSI handles multiple service orders from customers at the same time. Each logistics service order consists of multiple service processes, which are divided into two types, namely, the customized service process and the mass service process. The mass service process of customer *i* is conducted either it is integrated into the mass service process of customer *j*(*j* ≠ *i*) for a consolidated mass service operation or it is operated independently in its customized mode. The LSI analyses all these orders' service processes and their respective time requests and then inquires the FLSPs of each service process about its standard time for completing the service process. After that, the LSI needs to determine the optional CODP collection. Then the LSI sets up the scheduling optimization goals based on scheduling time, scheduling costs, and satisfaction degree of FPLSs, and chooses the optimal CODP position, obtaining the schedule plan.

**Figure 1:**
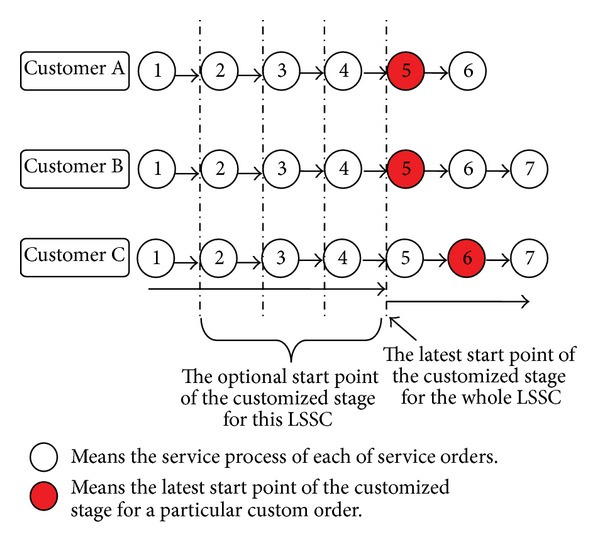
Schematic diagram of each order's operation process requirement in numerical example.

**Figure 2:**
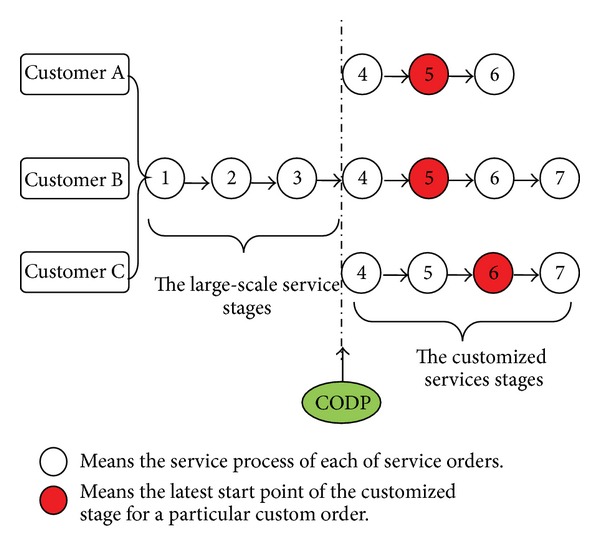
Take CODP is *k* = 4 for example, the orders' operation processes schematic diagram.

We use an example to demonstrate the scheduling problem. For example, as shown in Figure 1, there are three service orders from three customers that need to be scheduled by the LSI. The total amount of processes and the completion time of each order are different. However, some processes can be carried out together in the MC mode because of the similarity of the order contents. Assume that the total number of the service processes for these three orders is 6, 7, and 7, respectively, and these orders must be operated in the customized mode from the 5th, 5th, and 6th process according to customer requirements. Thus, without considering other constraints, the optional CODP in this numerical example is one of the elements in {2,3, 4,5}.  Figure 2 shows the LSSC order operation schematic when the CODP is located in Process 4.

**Figure 3:**
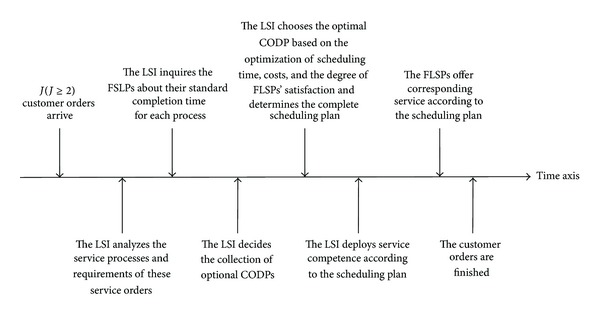
Sequence of events in the time scheduling of the LSSC.

The sequence of events in the LSSC time scheduling is shown in Figure 3, which consists of eight steps to complete the entire time scheduling.

1. *J*  customers' orders arrive and each order has its own customized requirements.
2. The LSI analyzes the service processes and requirements of each service order (and distinguishes the processes that can be operated in mass service mode from the ones that must be operated in the customized mode).
3. The LSI inquires the FSLPs about their standard completion time for each process.
4. The LSI decides the collection of possible CODPs.
5. Systematically considering three scheduling objectives, namely, the optimal total scheduling time, total costs of scheduling, and the satisfaction of FLSPs, the LSI establishes the time scheduling model and decides the optimal CODP and then obtains the complete specific scheduling plan.
6. According to the scheduling plan, FLSPs deploy their capability to guarantee that the completion time request to be met.
7. The FLSPs offer corresponding service according to the LSI's scheduling plan (maybe mass service or customization service).
8. Customer orders are finished.

In this study, by solving the model in step 5, the optimal CODP is determined and the scheduling plan is output. Based on this, we conduct a further analysis to explore the effect of changes in various scheduling parameters on the CODP positioning and the supply chain performance.  Table 1 shows the model notations.

### 3.2. Model Assumptions

**Assumption 1**

The LSI and FLSPs have established cooperative relationship and built the LSSC through contracts in strategic level. This paper focuses on how to allocate operation time for different service processes under circumstances where the cooperative relationship has been decided in order to maximize the supply chain scheduling performance. This paper does not consider the contract coordination problem in strategic level.

**Assumption 2**

Generally, the LSI possesses strong integration capability. It normally has a number of FLSPs and utilizes their capacities to meet different kinds of logistics service requests from customers. Therefore, we assume that logistics service capacities in each process are adequate and thus there is not any capacities constraint. Furthermore, we assume that the logistics service in each process is completed by one FLSP. The FLSPs can provide either mass service or customization service. But the cost of offering mass service is lower than that of offering customized service, and the normal service time to finish an order in the mass service mode is longer than that in the customized mode.

**Assumption 3**

In this paper, we assume that the normal operation time of each provides is settled and stationary, which is decided by the service order.

**Assumption 4**

Each scheduling task aims at only one set of customer orders and no new orders are added.

**Assumption 5**

In terms of time scheduling, additional service costs are incurred, regardless of whether the order is completed ahead of time or delayed. For the *j*th customer's order in the *i*th process, regardless of whether the order is completed ahead of time or delayed, the unit additional service cost *C*_*ij*_^ext^ is assumed to be the same.

**Assumption 6**

The normal service time refers to the usual time needed in completing a task using its FLSP capability. When the work is done in normal service time, the satisfaction of the FLSP is the highest. Conversely, if the FLSP works in abnormal time (e.g., time ahead of schedule or delayed), its satisfaction will decline.

**Assumption 7**

Based on the premise of meeting the customized requirements, each node (except at the beginning and the end of the logistics services) can be used as the CODP.

**Assumption 8**

No matter which step is chosen to be the CODP, switching operation (i.e., loading and unloading) time and cost exists. The operation time and unit cost are different because of the difference in CODP positioning.

### 3.3. Model Building

#### 3.3.1. Optimization Objectives of the Scheduling Model

When the LSI undertakes the time scheduling process of LSSC, multiple factors should be considered systematically. For example, the LSI needs to consider multiple requirements in terms of the service order's completion time and attempt to finish the service on time. The LSI cannot ignore the factor of the satisfaction of all FLSPs and must arrange transfer between each service process reasonably. To achieve the minimal scheduling cost, it is better to operate the service processes in mass service mode as more as possible. Therefore, to minimize the total cost of LSSC orders, minimize the difference between the expected time and actual time of completing the service orders, and maximize the satisfaction of all FLSPs, this paper establishes the LSSC time scheduling model based on the CODP. On the premise that the FLSPs' supply capacities are with uncertainty, the decision variable of the LSI to implement the LSSC time scheduling is the expected actual completion time of each FLSP. The FLSPs will adjust their completion time (compress or delay the order completion time) according to the difference between the expected time and their own normal completion time to reach the optimal scheduling performance.

(1)  Min⁡ Z1=f1+f2+f3+f4+f5,

(2) Min⁡ Z2=∑j=1J0|Tjexp⁡−Tj|Tjexp⁡×YjN,    Tj=∑i=1I0Tij+Tijext+Ti+Tiext,

(3) Max⁡ Z3   =(∑i=1k−1(1−|Ti−Tiexp⁡|Ti)(TiCiTiCi+|Tiext|Ciext)      +∑j=1J0 ∑i=kI0(1−|Tij−Tijexp⁡|Tij)      ×(TijCijTijCij+|Tijext|Cijext))    ×(k−1+(∑j=1J0(Ij−(k−1))))−1,

where

(4) [f(x)]+=max⁡⁡{0,f(x)}, f1=(1−ρk)∑i=1k−1(TiCi+|Tiext|Ciext)×Y, f2=∑j=1J0∑i=kIj(TijCij+|Tijext|Cijext)×Yj,f3=∑j=1J0FijHijYj,  i=k,f4=∑i=1k−1[Tiexp⁡−Ti−Tiext]+Pi×Y+∑j=1J0∑i=kIj[Tijexp⁡−Tij−Tijext]+Pij×Yj,f5=∑i=1k−1[Ti+Tiext−Tiexp⁡]+Di×Y+∑j=1J0∑i=kIj[Tijexp⁡−Tij−Tijext]+Dij×Yj.

In (1), the objective function *Z*_1_ is used to minimize the total scheduling cost of LSSC. *f*_1_ is the total cost of all costs incurred in the mass service processes. (1 − *ρk*) denotes the influence caused by the mass service effects. Along with the increase of mass service processes *k*, the unit cost of mass service decreases. *f*_2_ is the total cost of all costs incurred in the customized processes. *f*_3_ is the switching cost in CODP, which refers to the switching operation cost incurred when the mass service operation is completed and the customized operation is to be started. *f*_4_ is the penalty cost for the process, which is completed earlier than the expected time. *f*_5_ is the penalty cost for the process that is delayed compared with the expected completion time.

In (2), the objective function *Z*_2_ is used to complete all of these orders as punctually as possible to minimize the difference between the actual completion time and the expected completion time. Each weight is the proportion of each customer's order to the entire order.

In (3), the objective function *Z*_3_ is used to maximize the satisfaction of all FLSPs. The first part in the equation is the sum of satisfaction in the mass service operation stage, and the second part is the sum of satisfaction in the customized operation stage. Both parts are represented by the product of satisfaction in terms of time and satisfaction in terms of cost. (1 − (|*T*_*ij*_ − *T*_*ij*_^exp⁡^|/*T*_*ij*_)) indicates the proximity degree between the normal operation time and the order expected time set by the LSI for the *j*th FLSPs in the *i*th service process, which represents satisfaction in the time aspect. *T*_*ij*_ *C*_*ij*_/(*T*_*ij*_ *C*_*ij*_ + |*T*_*ij*_^ext^|*C*_*ij*_^ext^) indicates the proportion of the normal operation cost in the total cost of the *j*th customer's order in the *i*th service process, which represents the FLSP's satisfaction in the cost aspect. The denominator of (3) is the total amount of service processes. In the mass service stage, each service process carried out for all orders counts as one process; in the customized stage, each process for a single order counts as one process.

#### 3.3.2. Constraints of the Scheduling Model

The scheduling model needs some constraints, including the time constraint of the order completion time required by customers, the time constraint of the upstream process and downstream process, and the FLSPs' satisfaction degree constraint. Equations (5) to (10) are the constraints of the model:

(5) subject  to ∑i=1k−1(Ti+Tiext)         +∑i=kIj(Tij+Tijext)+Hkj≤Tjexp⁡(1+β),

(6)        Ti+1−≤Ti+Tiext−Tiexp⁡≤Ti+1+, i≤k−1,

(7)        T(i+1)j−≤Tij+Tijext−Tijexp⁡≤T(i+1)j+, i≥k,

(8)         (1−|Ti−Tiexp⁡|Ti)(TiCiTiCi+|Tiext|Ciext)        ≥Ui0, i≤k−1,

(9)        (1−|Tij−Tijexp⁡|Tij)(TijCijTijCij+|Tijext|Cijext)        ≥Uij0, i≥k,

(10)        θ=1J0∑j=1J0ΔKjKj≤ω.

Equation (5) is the time constraint of the customer requirements, which means that the completion time of each order cannot be longer than the maximum time delay range set by the corresponding customer. Equation (6) is the time constraint due to the connection relation between upstream and downstream in the mass service stage. This strong constraint must be obeyed because in the service provision process, the link in operation time exists between the upstream and downstream service processes. Equation (7) is the time constraint due to the connection between upstream and downstream in the customized stage. It is also a strong constraint. Equations (8) and (9) are the constraints of the FLSPs' satisfaction in the mass service stage and the customized stage, respectively, indicating that the satisfaction of each FLSP should be more than the lower limit that they can accept. Equation (10) is the constraint of order differences, which indicates that the actual order difference coefficient cannot be more than the one set by the LSI.

## 4. Model Solution

The solution of this LSSC time scheduling model is presented in this section. Generally, researches on scheduling are mainly categorized into two branches. One branch is aimed at studying the effectiveness and optimality in order to carry out quick scheduling in practice; another branch of the research focus is studying the influence of relative parameters on scheduling performance to find out the most influential ones and determine them reasonably. In Introduction, we points out that this paper mainly works on four aspects of deficiency in existing researches. Among them, the fourth kind of deficiency is discussing what scheduling parameters will affect scheduling results. Therefore, the model solution does not focus on the comparison or selection of different kinds of solution method instead it just chooses an appropriate solution method. In Section 4.1, this multiobjective model is simplified into a single-objective programming model. In Section 4.2, the genetic algorithm is used to solve this simplified model.

### 4.1. Simplifying the Multiobjective Programming Model

The LSSC time scheduling model has three objectives and six constraints. It is a typical multiobjective programming problem. The model solution cannot be simply carried out from the perspective of mathematical equations; its practical meaning should be considered as well. In the MCLS mode, cost is not the only consideration. Completing the order with the absolutely minimal cost is not necessary. It only needs to maintain the total cost of the LSSC within a certain range. To build and maintain a good relationship with FLSPs, LSIs are usually willing to give a certain amount of cost concession. Thus, the cost objective may be transferred to a new constraint (see, e.g., [43]). Taking these actual situations into account, we introduce a parameter called the relationship cost coefficient *c* into the model and use it to represent the cost augment limits. The new constraint of cost is shown in

(11) $$\begin{array}{c} Z_{1}<Z_{1}^{\min}\times \left(1+c\right). \end{array}$$

The new constraints are the original constraints combined with (11).

The original model then becomes a twin goal programming problem whose objectives are customer service time and satisfaction of FLSPs, that is, minimal service delivery time and maximum FLSP satisfaction. As some conflicts and incommensurability exist in each target in multiobjective decision-making problems, finding an absolute optimal solution is difficult. In terms of the solving method for multiobjective programming problems, many specialized solution methods can be used, such as the evaluation function method (e.g., linear weighting method, reference target law, and minimax method), target planning method, hierarchical sequence law, interactive planning, and subordinate function method. In this paper, referring to Liu et al. [6] and Liu et al. [43], we choose the most typical linear weighting method to solve our model. The objective function *Z*_2_ is used to find the minimal value of *Z*_2_, and the objective function *Z*_3_ is used to find the minimal value of *Z*_3_, both of which are dimensionless and *Z*_2_ ∈ (0,1), *Z*_3_ ∈ (0,1). After the mathematical transformation, the synthesized objective function is shown in

(12) $$\begin{array}{c} \max Z=K_{2}\times \left(1-Z_{2}\right)+K_{3}\times Z_{3}. \end{array}$$

In (12), *K*_2_ and *K*_3_, represent the weights of *Z*_2_ and *Z*_3_, respectively, which are determined through the linear weighting method (see, e.g., [43]). The new objective denotes the synthesized effect of the LSSC time scheduling, which is called the comprehensive performance of the LSSC.

The solving method in (12) also accords with the actual LSSC scheduling process. When implementing the multiorder scheduling process, the LSI needs to meet multicustomer completion requests as well as the satisfaction degree of FLSPs. If the LSI excessively emphasizes meeting the customers' time requests, its FLSPs may be unsatisfied which would lead to failure in the order completion. On the contrary, if the LSI immoderately emphasizes improving FLSPs' satisfaction, the service time of customer orders may not be guaranteed. Therefore, in the actual scheduling process, the LSI needs to keep a good balance between the objective weights of *Z*_2_ and *Z*_3_, trying to maximize the comprehensive performance objective of the supply chain. In the numerical analysis, Sections 5.6.1 and 5.6.2 will demonstrate the influence of different weights of objectives on the scheduling results.

Thus, the single-objective model is as follows:

(13)  max⁡   Z=K2×(1−Z2)+K3×Z3,

(14) subject  to ∑i=1k−1(Ti+Tiext)        +∑i=kIj(Tij+Tijext)+Hkj≤Tjexp⁡(1+β),

(15)        Ti+1−≤Ti+Tiext−Tiexp⁡≤Ti+1+, i≤k−1,

(16)        T(i+1)j−≤Tij+Tijext−Tijexp⁡≤T(i+1)j+, i≥k,

(17)       (1−|Ti−Tiexp⁡|Ti)(TiCiTiCi+|Tiext|Ciext)        ≥Ui0, i≤k−1,

(18)       (1−|Tij−Tijexp⁡|Tij)(TijCijTijCij+|Tijext|Cijext)         ≥Uij0, i≥k,

(19)        θ=1J0∑j=1J0ΔKjKj≤ω,

(20)        Z1≤Z1min⁡×(1+c).

### 4.2. Using the Genetic Algorithm to Solve the Multiobjective Programming Problem

The genetic algorithm is an effective method used to search for the optimal solution by simulating the natural selection process. As it uses multiple starting points to begin the search, it has a satisfactory global search capability. Since its overall search strategy and optimization search method are not dependent on gradient information or other aids but only the objective function, which affects search direction and corresponding fitness functions, the genetic algorithm offers an common framework to solve complicated system problems. The genetic algorithm does not depend on a specific field and is robust to the kinds of problems. Thus, genetic algorithm is widely used in many scientific fields today, such as combinatorial optimization (see, e.g., [44]), machine learning (see, e.g., [45]), signal processing (see, e.g., [46]), adaptive control, and artificial life (see, e.g., [47]). For the combinatorial optimization problem, the genetic algorithm is quite effective to solve NP problem, such as the production scheduling problem (see, e.g., [48–50]), travelling salesman problem (see, e.g., [51]), knapsack problem (see, e.g., [52]), bin packing problem (see, e.g., [53]), and graph partitioning problem (see, e.g., [54]).

Similar to the natural evolutionary processes, the computational process of the genetic algorithm is an iterative process that involves selection, crossover, and mutation processes. This kind of successive inheritance from individuals with high fitness to the next generation obtains the optimal solution at last. Our solution to the scheduling model does not focus on comparing or selecting the best method among different kinds of solution methods, and we just choose an appropriate method. Given the superiority of the genetic algorithm in solving programming problems and the successful application to scheduling problems (see, e.g., [48–50]), this paper uses the genetic algorithm to solve the proposed model.

## 5. Numerical Analysis

This section illustrates the validity of model by conducting a numerical analysis and then by exploring the influence of relevant parameters on the time scheduling results. We also give some effective recommendations for supply chain scheduling and optimization based on numerical analysis.  Section 5.1 presents the basic data of the numerical example.  Section 5.2 shows the scheduling results.  Section 5.3 discusses the influence of the time delay coefficient *β* of order completion on the scheduling results of the LSSC.  Section 5.4 presents the influence of the relationship cost coefficient *c* on the scheduling results. The influences of the order difference tolerance coefficient *w* and the mass service effects coefficient *ρ* obtained by LSI on the scheduling results are given in Sections 5.5 and 5.6, respectively.

### 5.1. Numerical Example Description and Basic Data

**Figure 4:**
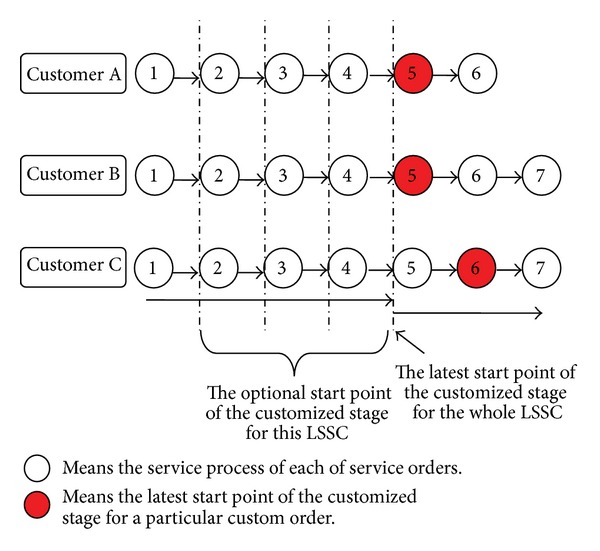
Schematic diagram of each order's operation process requirement in numerical example.

See from Figure 4 a two-echelon LSSC is assumed to consist of one LSI and many FLSPs. The LSI handles three customer service orders at the same time. The total process amount and completion time of each order are different. However, some processes can be carried out together in the MC mode because of the similarity of the order contents. The total number of service processes for these three orders is 6, 7, and 7, respectively. These orders must be operated in the customized mode from the 5th, 5th, and 6th processes according to customer requirements. Thus, without considering other constraints, the optional CODP in this numerical example is one of the elements in {2,3, 4,5}.

The parameter values used in our model are shown in the matrix below and in Table 2:

(21) $$\begin{array}{c} T=\left[\begin{array}{ccc} 7 & 6 & 6.5\\ 6 & 11 & 7\\ 10 & 9 & 9.5\\ 12 & 10 & 11\\ 6 & 7 & 7.5\\ 6 & 7 & 8\\ \text{—} & 7 & 10 \end{array}\right],\;\;C=\left[\begin{array}{ccc} 5 & 6 & 5.5\\ 7 & 8 & 8.5\\ 11 & 12 & 10.5\\ 15 & 18 & 14.5\\ 6 & 5 & 7.5\\ 7 & 6 & 7\\ \text{—} & 4 & 8 \end{array}\right],\\ F=\left[\begin{array}{ccc} 8 & 7 & 7\\ 8 & 6 & 6\\ 7 & 6 & 5\\ 5 & 4 & 4\\ 4 & 3 & 3\\ 4 & 3 & 2 \end{array}\right],\;\;H=\left[\begin{array}{ccc} 3 & 2.5 & 2.5\\ 3 & 2 & 2\\ 2.5 & 2 & 2\\ 4 & 4 & 3\\ 5 & 3 & 6\\ 5 & 6 & 7 \end{array}\right],\\ T^{\exp}=\left[\begin{array}{ccc} 9 & 7 & 9\\ 9 & 8 & 5\\ 8 & 9 & 13\\ 10 & 13 & 10\\ 8 & 6 & 9\\ 5 & 8 & 12\\ \text{—} & 9 & 12 \end{array}\right],\;\;P=\left[\begin{array}{ccc} 4 & 6 & 6\\ 6 & 8 & 6\\ 8 & 8 & 10\\ 10 & 7 & 5\\ 6 & 7 & 5\\ 6 & 8 & 7\\ \text{—} & 4 & 5 \end{array}\right],\\ D=\left[\begin{array}{ccc} 2 & 4 & 4\\ 5 & 4 & 4\\ 7 & 8 & 9\\ 8 & 8 & 5\\ 5 & 4 & 5\\ 6 & 5 & 4\\ \text{—} & 5 & 3 \end{array}\right],\;\;C^{\text{ext}}=\left[\begin{array}{ccc} 7 & 8 & 7\\ 10 & 9 & 10\\ 13 & 14 & 13\\ 18 & 20 & 16\\ 8 & 7 & 9\\ 9 & 8 & 9\\ \text{—} & 6 & 10 \end{array}\right],\\ U=\left[\begin{array}{ccc} 0.6 & 0.5 & 0.55\\ 0.5 & 0.6 & 0.55\\ 0.4 & 0.5 & 0.55\\ 0.4 & 0.4 & 0.65\\ 0.5 & 0.45 & 0.5\\ 0.6 & 0.6 & 0.5\\ \text{—} & 0.5 & 0.65 \end{array}\right],\\ T_{\left(i+1\right),j}^{-}=\left[\begin{array}{ccc} \text{—} & \text{—} & \text{—}\\ -3 & -3 & -5\\ -4 & -4 & -3\\ -5 & -2.5 & -5\\ -5 & -4 & -3\\ -4 & -5 & -2.5\\ \text{—} & -2.5 & -4 \end{array}\right],\\ T_{\left(i+1\right),j}^{+}=\left[\begin{array}{ccc} \text{—} & \text{—} & \text{—}\\ 3 & 4 & 5\\ 4 & 5.5 & 3\\ 3 & 4 & 4\\ 3.5 & 5 & 2\\ 5 & 3.5 & 4.5\\ \text{—} & 5 & 3 \end{array}\right]. \end{array}$$

Considering its practical significance, our numerical example requires that

(22) $$\begin{array}{c} \left|T_{ij}^{\text{ext}}\right|\leq 0.3T_{ij},\;\;\left|T_{i}^{\text{ext}}\right|\leq 0.3T_{i}. \end{array}$$

As shown in (22), in practical scheduling, FLSP's completion time range of a certain service process is generally proportional to the normal working time, regardless of whether the operation time is delayed or ahead of schedule. In this paper, we assume that a certain service process is delayed or completed in advance by not more than 0.3 times the normal working time.

### 5.2. Numerical Example Results

In the model solution, we use the genetic algorithm and Matlab 7.8 software to solve the problem. Assuming that the genetic population is 800, the hereditary algebra is 800, the delay coefficient of the order completion time is *β* = 0.05, the relationship cost coefficient is *c* = 0.2, the order difference tolerance coefficient is *w* = 0.5, and the mass service effect coefficient obtained by LSI is *ρ* = 0.1, the calculation result is as follows.

The optimal solution is *Z* = 0.9324, and *k* = 4 is the optimal CODP. Thus, the first three processes are part of the mass service operation stage, and the remaining ones are part of the customized operation stage. The corresponding scheduling results are as follows:

 Mass service operation stage:
  (23) $$\begin{array}{c} \left[\begin{array}{ccc} T_{1}^{\text{ext}} & T_{2}^{\text{ext}} & T_{3}^{\text{ext}} \end{array}\right]=\left[\begin{array}{ccc} 0.0759 & 0.2400 & -0.0929 \end{array}\right]. \end{array}$$
 Customized operation stage:
  (24) $$\begin{array}{c} \left[\begin{array}{ccc} T_{41}^{\text{ext}} & T_{42}^{\text{ext}} & T_{43}^{\text{ext}}\\ T_{51}^{\text{ext}} & T_{52}^{\text{ext}} & T_{53}^{\text{ext}}\\ T_{61}^{\text{ext}} & T_{62}^{\text{ext}} & T_{63}^{\text{ext}}\\ \text{—} & T_{72}^{\text{ext}} & T_{73}^{\text{ext}} \end{array}\right]=\left[\begin{array}{ccc} 1.4879 & 0.7492 & -0.9107\\ 1.4837 & 0.0022 & 0.0113\\ 0.6990 & 0.0155 & 0.0102\\ \text{—} & 0.0000 & -0.8458 \end{array}\right]. \end{array}$$

The detailed time scheduling results are shown in Table 3.

### 5.3. Effect of *β* on the Scheduling Performance of the LSSC

In the MCLS, responding quickly to customize requirements (including time requirement) is an important objective of time scheduling. Thus, customers' required completion time of a service order may change, and time compression or delay requirement is possible, demanding a certain degree of time flexibility in scheduling from the LSI. In model building, *β*_*i*_ < 0 means that the service order needs to be finished ahead of time; accordingly, *β*_*i*_ > 0 means that the service time needs to be delayed. Clearly, the permitted order completion time is directly related to the FLSPs' difficulty in completing the order. This order completion time directly affects the costs of these FLSPs.

**Figure 5:**
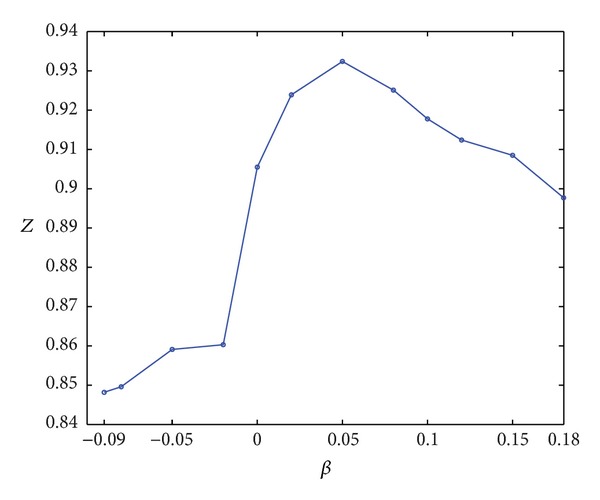
Curve of *Z* changed with *β*.

In this section, we discuss the influence of the delay (or compression) coefficient of order completion time *β* on the three objective functions, namely, total satisfaction of FLSPs, service delivery punctuality, and total cost of LSI. Keeping the model parameters unchanged and changing only the value of *β*, we calculate the corresponding results for *Z*. These results are shown in Table 4. By plotting the data in Table 4, we obtain Figure 5.

Based on Table 4 and Figure 5, the following conclusions can be obtained.

**Figure 6:**
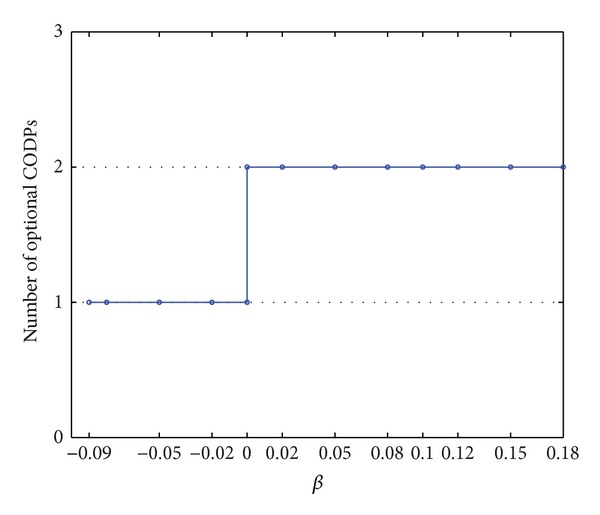
Curve of the number of optional CODPs with *β*.

1. With the increase in *β* (from negative to positive), *Z* first increases and then decreases, which means that a reasonable positive tolerance coefficient contributes to achieving the maximal value of comprehensive performance (i.e., when *β* = 0.05, comprehensive performance reaches the maximum *Z* = 0.93). Conversely, if *β* is negative, the maximal value of comprehensive performance cannot be reached. Moreover, a smaller time delay tolerance coefficient (i.e., the service should be operated in time compression) results in poorer comprehensive performance. Therefore, in practice, comprehensive performance can deteriorate when customers request shortening the order completion time of FLSP.
2. *Z* significantly increases when *β* changes from being negative to positive. If the FLSP operates in the case in which the operation time is ahead of schedule and the LSI provides some lag time, then the comprehensive performance of the LSSC significantly improves more than that in the case in which the FLSP operates in a time delay state.
3. If *β* < −0.09, the model has no solution, which means that the LSSC cannot operate in time compression without limit. Furthermore, the LSSC scheduling has certain characteristics, and the order cannot be completed as early as the customer wants it. When a time delay is set by customers, comprehensive performance will not increase all the time, as the increase is followed by a decrease after a certain inflection point. Comprehensive performance will not increase to infinity even when the customer permits a delay in the order completion time. On the contrary, an optimal delay coefficient of order completion time exists.
4. When *β* changes from being negative to positive, the range of optional CODP changes from either 4 or 5 to 4 only;that is, the number of optional CODPs is reduced from 2 to 1 (Figure 6). Given the reinforcement to shorten the completion time, the optional CODP range and supply chain flexibility are reduced. In this case, LSI has to sacrifice cost and increase customization to meet the time requirement. Therefore, a reasonable *β* should be chosen to guarantee an appropriate degree of customization in practice.

### 5.4. Effect of *c* on the Scheduling Result of the LSSC

In the model solution, the relationship cost coefficient *c* is introduced into the solution approach, and the cost objective of the LSI changes to a new constraint. Therefore, pursuing the minimal cost objective is not necessary, but the cost should be kept within a reasonable range. Generally, the relationship cost coefficient *c* is decided by the LSI, and its size directly influences FLSP satisfaction and LSSC comprehensive performance. In this section, we explore the effect of the relationship cost coefficient of LSI on comprehensive performance.

By changing only the value of *c* and keeping other parameters unchanged, the corresponding *Z* and Z* can be obtained (see Table 5) to explore the relationship between *c* and LSSC comprehensive performance (denoted by *Z* and Z*). In this numerical example, LSSC can operate smoothly when CODP is positioned at *k* = 4 or *k* = 5, but their corresponding comprehensive performances are different. Specifically, the comprehensive performance of *k* = 4 is better than that of *k* = 5.

**Figure 7:**
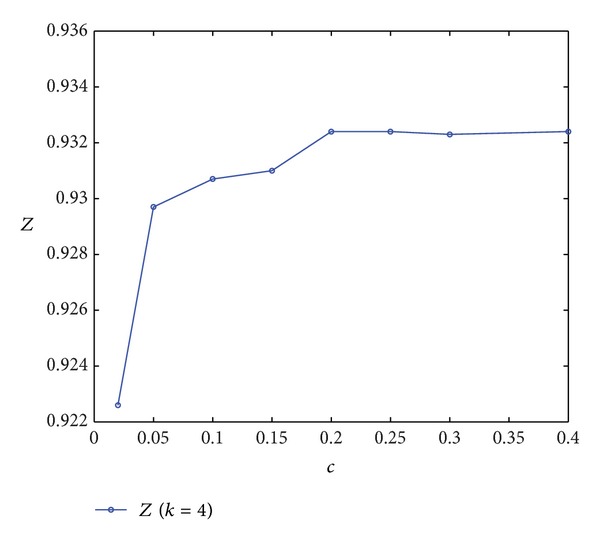
Curve of *Z* varied with *c*.

By plotting the data in Table 5, we obtain Figure 7.

Figure 7 clearly shows that *Z* increases with the increase in *c* and ultimately tends to be stable. The comprehensive performance of LSSC increases with the increase in the relationship cost coefficient of the LSI and remains stable after reaching a certain value. When *c* increases from 0 to 0.05, the slope of the curve is relatively large. Afterwards, the growth in comprehensive performance slows down with the increase in *c* and ultimately stabilizes at the value 0.9324. The implication is that, after *c* increases to a certain level, a continued increase in cost will not contribute to the improvement of the comprehensive performance of the LSSC. Moreover, the improvement in supply chain comprehensive performance caused by the increase in the cost relationship coefficient has certain limitations.

### 5.5. Effect of *w* on the Scheduling Results

In this paper,  *θ* = (1/*J*_0_)∑_*j*=1_^*J*_0_^(Δ*K*_*j*_/*K*_*j*_) denotes the order difference coefficient. *w* is the order difference tolerance coefficient of LSI, which is decided by LSI. In this section, we explore the influence of the LSI's order difference tolerance coefficient *w* on the scheduling results.

This section discusses the influence of *w* on the scheduling results by changing only *w* and keeping *β* = 0.05, *ρ* = 0.1, and *c* = 0.2 unchanged. Results in Table 6 were obtained using Matlab 7.8. software.

**Figure 8:**
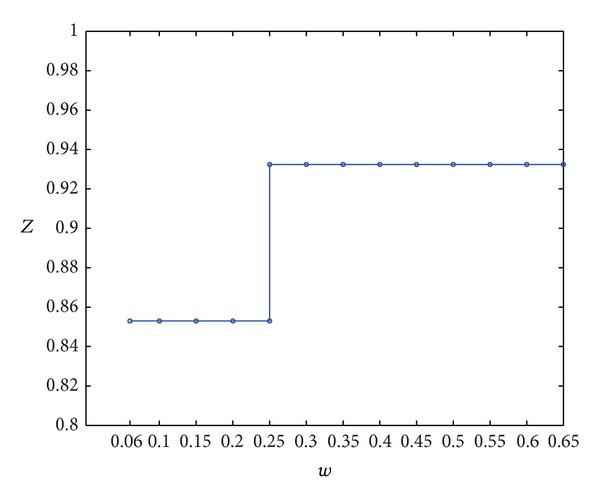
Curve of *Z* varied with *w*.

**Figure 9:**
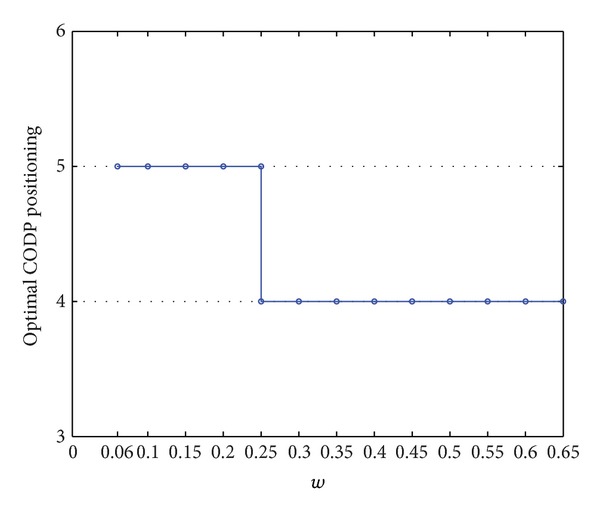
Curve of the optimal CODP varied with *w*.

In Figure 8, the comprehensive performance *Z* shows a step-shaped growth along with the increase in *w*. Theoretical analysis of the model shows that the main role of *w* is to restrict the range of optional CODP. In our numerical examples, the variation in *w* mainly leads to changes in the selectable range of CODP in set {4,5}, considering other constraints. In Figure 9, if *w* is too small (i.e., *w* < 0.06 in this example), no solution is obtained, indicating that LSSC cannot operate in this case. When 0.06 < *w* < 0.25, *k* = 5 leads to optimal comprehensive performance Z* = 0.8530; when *w* = 0.25, *k* = 4 leads to optimal comprehensive performance Z* = 0.9324; when *w* > 0.25, the increase in *w* no longer increases comprehensive performance. Thus, LSI must set a reasonable value for the order difference tolerance coefficient *w*. If *w* is too small (i.e., *w* < 0.06 in this example), the supply chain will not work at all. LSI should make its order difference tolerance coefficient *w* as large as possible to accumulate more customer orders and obtain mass service effects. At the same time, *w* should be kept in a proper range, as a too large *w* is not beneficial to increasing the comprehensive performance of LSSC.

### 5.6. Effect of the Mass Service Effect Coefficient *ρ* Obtained by the LSI on the Scheduling Results

Success of the MC mode lies in reducing total service cost by realizing the mass service effects in the premise of meeting the customized requirements. In this section, we discuss the influence of the mass service effect coefficient *ρ* obtained by the LSI on the scheduling results of the LSSC.

The numerical analysis in previous sections mainly focuses on the performance of time scheduling and not much on the cost objective. We regard cost objective as a new constraint. However, to discuss the influence of *ρ* on the scheduling results in this section, *ρ* will influence the total cost of the LSSC. Therefore, we consider three subtargets, namely, cost objective, punctual service delivery objective, and satisfaction of all FLSPs, in the overall objective at the same time. The influence of *ρ* obtained by the LSI is discussed by assigning different weights to these three subtargets.

#### 5.6.1. Effect of the Variation of *ρ* on the Scheduling Results When the Weights of *Z*_1_, *Z*_2_, and *Z*_3_ are the Same

The same weights are assigned to *Z*_1_, *Z*_2_, and *Z*_3_, that is; their weights are all 1/3. Each of these objective functions should be normalized before synthesis. The minimum value of *Z*_1_ (denoted by *Z*_1_^min⁡^) should be calculated when the objective functions *Z*_2_ and *Z*_3_ are considered. The resulting overall objective function is shown in

(25) $$\begin{array}{c} \max\;\;Z^{\prime }=\frac{1}{3}\times \frac{Z_{1}^{\min}}{Z_{1}}+\frac{1}{3}\times \left(1-Z_{2}\right)+\frac{1}{3}\times Z_{3}. \end{array}$$

The other eight constraints, namely (14) to (19) and (22), remain unchanged.

The value of *Z*_1_^min⁡^ mentioned above is the minimum obtained by changing the selectable range of CODP (i.e., *k*) and the mass service effect coefficient *ρ*. Based on Matlab 7.8 calculations, *Z*_1_^min⁡^ = 3088.2 and *k* = 5 at that point. Therefore, the first four processes are part of the mass service stage, and the remaining ones are part of the customized stage. The specific scheduling results are as follows:

Mass service stage:

(26) [T1extT2extT3extT4ext]  =[0.6319−2.6886−1.1872−2.0337].

Customized stage:

(27) $$\begin{array}{c} \left[\begin{array}{ccc} T_{51}^{\text{ext}} & T_{52}^{\text{ext}} & T_{53}^{\text{ext}}\\ T_{61}^{\text{ext}} & T_{62}^{\text{ext}} & T_{63}^{\text{ext}}\\ \text{—} & T_{72}^{\text{ext}} & T_{73}^{\text{ext}} \end{array}\right]=\left[\begin{array}{ccc} 1.4994 & -0.0008 & -0.8043\\ 0.0000 & 0.9926 & 0.0011\\ \text{—} & 0.0000 & -2.2193 \end{array}\right]. \end{array}$$

Keeping other parameters such as *β* = 0.05 and *w* = 0.5 unchanged, *ρ* is changed to obtain the corresponding scheduling results using Matlab 7.8 software. Different CODP positions (i.e., different *k*) produce different scheduling results. The model has a solution only when *k* = 4 and *k* = 5. Specific results are shown in Table 7.

**Figure 10:**
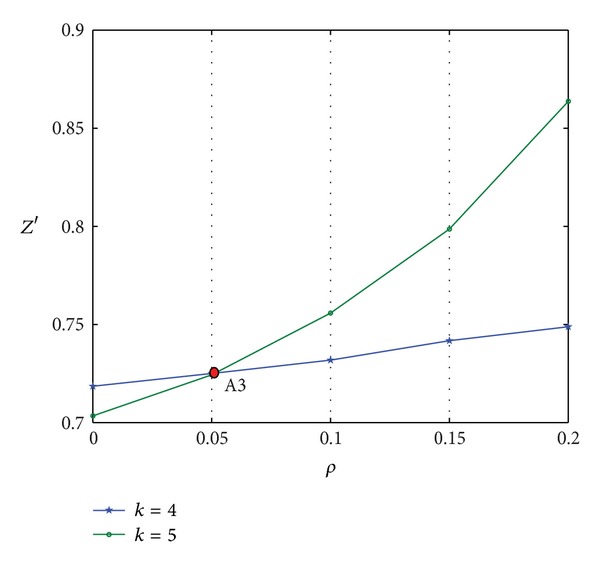
Curve of *Z*′ varied with *ρ*.

The data in Table 7 are plotted in Figure 10.

**Figure 11:**
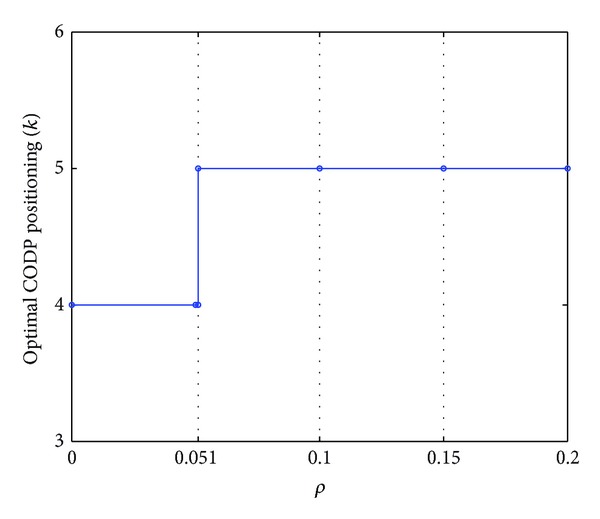
Curve of optimal CODP varied with *ρ*.

Figures 10 and 11 indicate the following.

1. The comprehensive scheduling performance of LSSC increases with the increase in *ρ*, no matter where the CODP is positioned.
2. The two curves in Figure 10 show that when the CODP varies, the comprehensive performance of LSSC also varies. When *ρ* is relatively small (*ρ* < 0.051), *k* = 5 improves the comprehensive performance of the supply chain. This performance is improved by *k* = 4 when *ρ* is relatively large (*ρ* > 0.051). The A3 point is the change point for the optimal CODP.
3. As the mass service effect is the characteristic MCLS, in practice, LSI should choose a reasonable CODP according to *ρ*. That is, the factor of *ρ* should be considered in the CODP decision.

#### 5.6.2. Effect of the Variation in *ρ* on the Scheduling Results When the Weights of *Z*_1_, *Z*_2_, and *Z*_3_, Are Different

Generally, total cost and service delivery punctuality are the most important objectives in scheduling for the LSI. Different LSIs have different attitudes toward the relative importance of cost and service delivery punctuality, thus affecting scheduling results. This section provides an in-depth discussion of the effects of the different objective weights on the scheduling results of the LSSC.

*k*_1_, *k*_2_, and *k*_3_ refer to the weights of cost objective, punctual service delivery objective, and FLSP satisfaction objective, respectively, in the synthesized objective function. In this section, we introduce the coefficient of weight difference degree *R* to represent the degree of difference in attitudes toward the relative importance of cost and service delivery punctuality. We let *R* = *k*_1_/*k*_2_. When *R* > 1, the LSI places more focus on cost objective; when *R* < 1, the punctual service delivery objective receives more attention. In the numerical simulation, *k*_3_ = 1/3 is unchanged, *R* is assigned different values, and *ρ* varies with each value of *R*. In what follows, we discuss the influence of the variation of *ρ* on the scheduling results.

(*1) Effect of the Variation of ρ on the Scheduling Results When R* = 3. The results are shown in Table 8.

**Figure 12:**
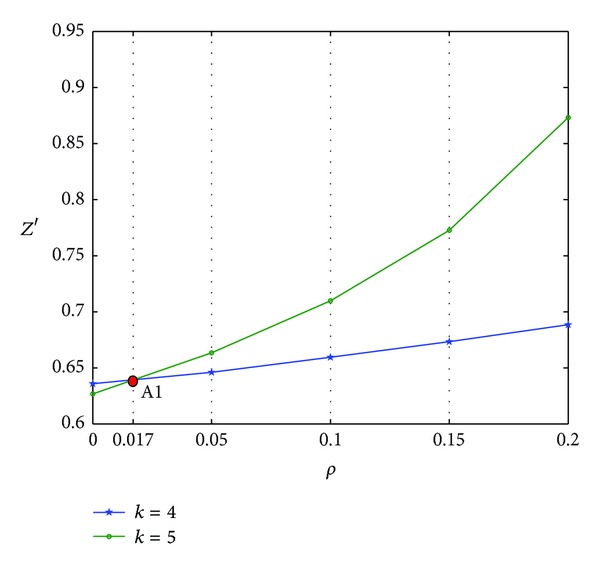
*Z*′ varied with *ρ*  (*R* = 3).

**Figure 13:**
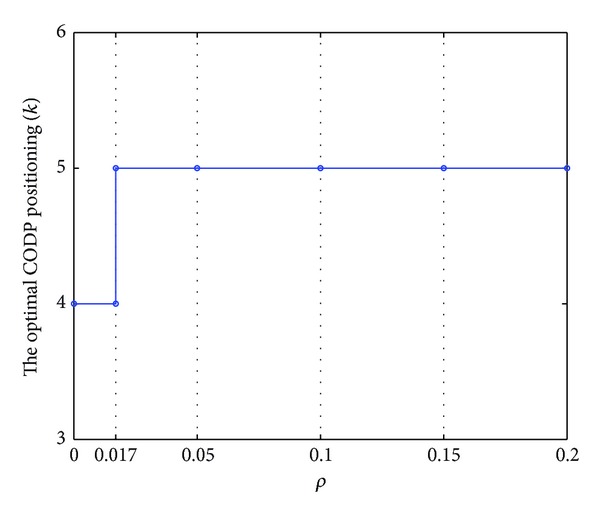
Optimal CODP varied with *ρ*  (*R* = 3).

As shown in Figures 12 and 13, *k* = 4 or 5 is the optimal CODP. The comprehensive performance of scheduling *Z*′ increases with the increase in *ρ*. A1 is the intersection of the two curves, where *ρ* = 0.017. A1 point is the changing point where the optimal CODP changes from *k* = 4 to *k* = 5.

(*2) Effect of the Variation of ρ on the Scheduling Results When R Assumes Other Values.* Table 9 shows the influence of *ρ* on the scheduling results when *R* assumes other values.

**Figure 14:**
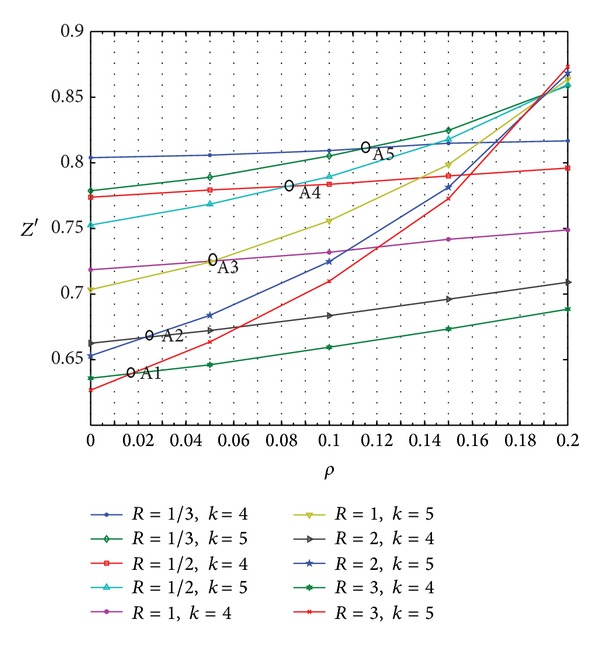
Effect of *ρ* on the scheduling results when *R* assumes different values.

In Table 9, comprehensive performance *Z*′ increases with *ρ*, regardless of the value of *R*. The LSSC can choose different locations for the CODP (in this example, either *k* = 4 or *k* = 5). The comprehensive performance of LSSC scheduling differs when the CODP varies. When *ρ* is relatively small, *k* = 4 is the optimal position. When *ρ* is relatively large, *k* = 5 is better. For different values of *R*, a corresponding threshold for *ρ* exists at which the optimal CODP changes from *k* = 4 to *k* = 5. The data are plotted in Table 9 to better present the variation in these thresholds, as shown in Figure 14.

As shown in Figure 14, the effects of *ρ* on the scheduling results differ because of the different CODPs and different preferences of the LSI. However, Figure 14 shows that all of these curves tend to increase with *ρ*. Comparatively, the combined condition of *k* = 5, *R* = 3 makes the slope of this curve the largest, indicating that, in this case, *Z*′ increases the fastest with *ρ*.

**Figure 15:**
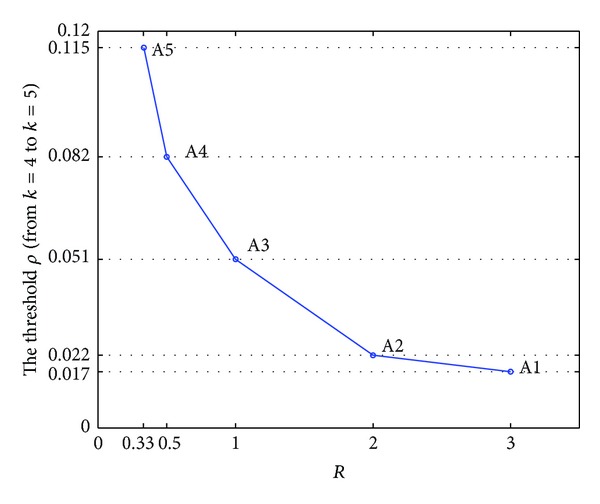
Curve of the thresholds of *ρ* at which the optimal CODP changes from *k* = 4 to *k* = 5.

Figure 15 presents the variation thresholds of *ρ* at which the optimal CODP changes from *k* = 4 to *k* = 5 at different values of *R*. According to Figure 15, we obtain the following conclusions.

1. The threshold of *ρ* shows that the change in the optimal CODP increases with the decline in *R*. This threshold indicates that the more the LSI pays attention to the cost goal, the smaller the threshold of *ρ* becomes; the more the LSI pays attention to the punctual service delivery goal, the larger the threshold of *ρ* becomes. Moreover, the more the LSI pays attention to the cost goal, the greater the motivation to enlarge the CODP position is (from *k* = 4 to *k* = 5), indicating that LSI can change its CODP position when the mass service effect coefficient is relatively small.
2. When other conditions are the same, customers are better off choosing LSI, whose mass service effect coefficient is relatively large, to obtain a higher level of customization.
3. Figure 15 shows that the scope of this curve tends to be zero with the increase in *R*, indicating that the difference in these thresholds of *ρ* tends to decrease with the increase in *R*. For example, when *R* increases from 1/3 to 1/2, the threshold of *ρ* changes from 0.115 to 0.082, with a difference of 0.033. When *R* increases from 2 to 3, the threshold of *ρ* changes from 0.022 to 0.017, with a difference of only 0.005. Thus, for the LSI whose mass service effect coefficient is relatively small, improving *R* contributes to obtaining scale cost effects. By contrast, for the LSI whose mass service effect coefficient *ρ* is relatively large, the mass service effects are evident because of the relatively large value of *ρ*. Therefore, choosing a relatively small value of *R* can either obtain good mass service effects or improve service delivery punctuality. In this case, a relatively good level of scheduling performance of LSSC can still be reached.

## 6. Main Conclusions and Management Insights

In this section, we present the main conclusions of this research and explain related insights for researchers. We also discuss management insights for LSI and propose related recommendations for time scheduling decisions.

### 6.1. Main Conclusions Derived from the Scheduling Model

The following conclusions are based on the previous analysis.

(1)  *Z* first increases and then decreases with the delay coefficient of order completion time *β* (from negative to positive). The more the order completion time needs to be compressed, the worse the comprehensive performance of the LSSC becomes. When *β* is reduced to a certain extent, the model has no feasible solution, which means that the LSSC cannot operate with too much time compression. When a delay in the order completion time is allowed (*β* > 0), the increase in *β* improves comprehensive performance. However, comprehensive performance deteriorates after *β* increases to a certain extent because of the trade-off relationship between the objective functions *Z*_2_ and *Z*_3_, which cannot be optimal at the same time.

(2) Numerical analysis shows that when the value of *β* changes from negative to positive, the selectable range of the CODP is reduced from either *k* = 4 or 5 to only *k* = 4, and correspondingly the feasible position of the CODP is reduced from 2 to 1. This reduction indicates that the optimal CODP position tends to move forward with the stronger requirement to complete the customer order ahead of time, thus reducing the mass service processes and increasing customization. Furthermore, the selectable range of the CODP position and the flexibility of the LSSC decrease with the stronger requirement to complete the customer order ahead of time. The time scheduling requirements need to be satisfied by sacrificing cost and increasing customization. Therefore, in practice, a reasonable *β* must be chosen to guarantee reasonable customization.

(3) Comprehensive performance *Z* increases and then tends to be stable with the increase in the cost relationship coefficient. Thus, after reaching a certain level, increasing *c* no longer contributes to improving the comprehensive performance of the LSSC. Improving in supply chain comprehensive performance caused by the increase in the cost relationship coefficient has certain limitations.

(4) Comprehensive performance *Z* shows step-shaped growth with the increase in the order difference tolerance coefficient *w* of the LSI. However, after *w* reaches a certain value, comprehensive performance remains stable. If *w* is too small (i.e., *w* < 0.06 in this example), the supply chain will not work. Thus, the coefficient *w* cannot be too small, and LSI needs to enhance its order difference tolerance coefficient *w* to achieve better scheduling performance.

(5) Whether the weights of *Z*_1_, *Z*_2_, and *Z*_3_ are the same or not, the comprehensive performance of the LSSC improves with the increase in *ρ*. With same value of *ρ*, comprehensive performance varies when the CODP locations are different. Therefore, the LSI should choose FLSPs whose mass service effect coefficient is relatively large to obtain more profits.

(6) The preference for the relative concern degree of the LSI for cost and service delivery punctuality leads to differences in the scheduling performance of the LSSC. Using *R* to represent the ratio of the weights of the cost objective and the punctual service delivery objective, we can find that the threshold of *ρ*, which reflects changes in the optimal CODP, decreases with the increase in *R*. Therefore, the more the LSI pays attention to the cost goal, the smaller the threshold of *ρ* becomes; the more the LSI pays attention to the punctual service delivery goal, the larger the threshold of *ρ* becomes. When other conditions are the same, customers are better off choosing the LSI whose mass service effect coefficient is relatively large to obtain higher level of customization.

(7) The differences in the thresholds of *ρ* tend to decrease with the decrease in *R*. Thus, for the LSI whose mass service effect coefficient is relatively small, improving the weight difference *R* contributes to the attainment of scale cost effects. Conversely, for the LSI whose mass service effect coefficient is relatively large, reducing the value of *R* can either obtain good mass service effects or improve service delivery punctuality, ultimately optimizing comprehensive performance.

### 6.2. Implications for Researchers

This study establishes the LSSC time scheduling model based on the CODP and analyzes the time scheduling problem in the MCLS environment in detail to minimize the total LSSC operation cost, reduce the gap between the expected and the actual time of completing service orders, and maximize the satisfaction of all FLSPs. This study provides theoretical basis for further studies on the scheduling method and performance optimization method of LSSCs in the MCLS environment. For example, we find that both the relationship cost coefficient *c* and order difference tolerance coefficient *w* greatly affect the comprehensive performance of the LSSC. The different preferences for the relative concern degree of LSI on cost and service delivery punctuality lead to differences in the scheduling performance of LSSC. The comprehensive performance of the LSSC improves with the increase in the mass service effect coefficient obtained by the LSI. These important conclusions provide some basis for further in-depth studies on time scheduling models. Empirical research can be conducted on the relationship between the comprehensive performance of supply chain scheduling and the factors involved in scheduling. The best decision-making method for the time scheduling process of LSSC can be further discussed. In short, this study provides the necessary theoretical foundation for further development of both theoretical and empirical studies on LSSC scheduling in the MCLS environment.

### 6.3. Implications for Managers

The conclusions presented in this paper can serve as reference for the participants in LSSC, especially LSI. Results show that through the reasonably designed time scheduling decision and positioning of the optimal CODP, the LSI can maximize comprehensive performance and obtain optimal scheduling results.

We offer several management insights for LSI. First, the LSI can use the strategy of increasing the relationship cost coefficient to improve supply chain performance, but note that this kind of improvement is limited. Second, the LSI must keep a reasonable order difference tolerance coefficient and increase this coefficient to improve performance. Third, regardless of the relative concern degree of LSI for cost and service delivery punctuality, the comprehensive performance of the LSSC improves with the increase in *ρ*. Therefore, the LSI should choose FLSPs whose mass service effect coefficient is relatively large to obtain better supply chain performance. Fourth, if the mass service effect coefficient obtained by the LSI is relatively small, then the LSI should pay more attention to the cost goal and relax the punctual service delivery goal. Conversely, if the mass service effect coefficient of the FLSP chosen by the LSI is relatively large, then the LSI should pay more attention to the punctual service delivery goal and relax its concerns over the cost goal.

## 7. Research Limitations and Directions for Future Research

Based on the literature review of the existing scheduling model of the LSSC, this study established the LSSC time scheduling model based on the CODP to minimize the total operational cost for orders in LSSC, minimize the gap between the expected and actual time of completing service orders, and maximize the satisfaction of FLSPs. Numerical analysis was conducted using Matlab 7.8 software. The influence of parameters, such as delay coefficient of order completion time, relationship cost coefficient, and relative concern degree of LSI for cost and service delivery punctuality, on LSSC comprehensive performance was discussed. Through this model, an optimal scheduling plan can be developed. The LSI should set appropriate scheduling parameters to obtain the best scheduling performance of the LSSC.

However, this paper has several limitations. For example, the model solution and analysis are only in accordance with a real numerical example and are thus not representative of all situations in reality. Moreover, the assumption is that the scheduling operation only aims at a set of customer orders and does not consider new arrival orders. In practice, several orders may arrive in succession. Furthermore, our model does not consider the uncertainty of the capacity of FLSPs. In practice, the service-providing capacity of FLSP may be full of uncertainty because of external influence. In the future, a time scheduling model that considers such uncertainty can be established.

## Figures and Tables

**Table 1:** Notations for the model.

Notations	Description
*c*	The relationship cost coefficient of the LSI.
*C*_*i*_	The normal service cost per unit time per unit quantity of the *i*th process in offering mass operation.
*C*_*i*_^ext^	The extra service cost per unit time per unit quantity of the *i*th process in offering mass operation.
*C*_*ij*_	The normal service cost per unit time per unit quantity of the *i*th process in offering customized operation for the *j*th customer.
*D*_*i*_	In mass processes, the penalty cost per unit time per unit quantity of the *i*th process, if the order completion time is delayed.
*D*_*ij*_	In customized processes, the penalty cost per unit time per unit quantity of the *i*th process in offering customized operation for the *j*th customer, if the order completion time is delayed.
*F*_*ij*_	If the *i*th process is chosen to be CODP, the cost unit time per unit quantity needs to pay for the *j*th customer order.
*H*_*ij*_	The operation and switching time in the *i*th service process for the *j*th customer order due to the switching from mass to customized operation. It varies with *i* and *j* and is decided by the technology capability of the FLSP in the *i*th process.
*I*_*j*_	The total amount of service processes of the *j*th customer order.
*k*	The optimal CODP for all the orders.
*K*_*j*_	The latest possible CODP for the *j*th customer order (the latest point where customized operation must begin).
*k*_*t*_	Weight of the objective function *Z*_*t*_ in *Z*′, *t* = 1,2, 3.
*K*_*t*_	Weight of the objective function *Z*_*t*_ in *Z*, *t* = 2,3.
Δ*K*_*j*_	The difference between the latest possible CODP for the *j*th customer order and the optimal CODP for all the orders, Δ*K*_*j*_ = *K*_*j*_ − *k*.
*L*_*j*_	The total amount of processes in the *j*th customer order.
*N*	The total amount of customers' orders.
*P*_*i*_	In mass processes, the penalty cost per unit time per unit quantity of the *i*th process, if order is finished ahead of the expected time.
*P*_*ij*_	In customized processes, the penalty cost per unit time per unit quantity of the *i*th process in offering customized operation for the *j*th customer, if order is finished ahead of the expected time.
*R*	The coefficient of relative concern degree of LSI for cost and service delivery punctuality.
*T*_*i*_	For a certain order set, the normal service time of the *i*th process in offering mass operation, *i* = 1,2, 3,…, *I*_0_, the same below.
*T*_*ij*_	The normal service time of the *i*th service process in offering customized operation for the *j*th customer, *j* = 1,2, 3,…, *J*_0_, the same below.
*T*_*i*_^exp⁡^	The expected operation time of FLSPs for the *i*th service process set by LSI in offering mass operation.
*T*_*j*_^exp⁡^	The expected completion time of an order set by LSI's *j*th customer.
*T*_*ij*_^exp⁡^	The expected operation time of FLSPs for the *i*th service process in offering customized operation for the *j*th customer set by LSI.
*T*_*i*+1_^+^	In mass processes, the upper limit of the time delay incurred in the (*i* − 1)th service process which could be endured by the *i*th service process. It is determined by the rigid requirement caused by upstream and downstream operations of LSSC.
*T*_*i*+1_^−^	In mass processes, the upper limit of the time ahead of schedule incurred in the (*i* − 1)th service process which could be endured by the *i*th service process, which is determined by the rigid requirement caused by upstream and downstream operations of LSSC.
*T*_(*i*+1),*j*_^+^	In customized processes, for the *j*th customer order, the upper limit of the time delay incurred in the (*i* − 1)th service process which could be endured by the *i*th service process, which is determined by the rigid requirement caused by upstream and downstream operations of LSSC.
*T*_(*i*+1),*j*_^−^	In customized processes, for the *j*th customer order, the upper limit of the time ahead of schedule incurred in the (*i* − 1)th service process which could be endured by the *i*th service process, which is determined by the rigid requirement caused by upstream and downstream operations of LSSC.
*U*_*i*_^0^	The lower limit of the satisfaction degree of the *i*th mass process.
*U*_*ij*_^0^	The lower limit of the satisfaction degree of the *i*th customized process of the *j*th customer order.
*Y*_*j*_	The number of customized orders of the *j*th customer.
*Z*_1_	The total cost of LSSC.
*Z*_2_	The closeness degree of the actual order completion time and the expected one set by its customer.
*Z*_3_	The average satisfaction of all processes in LSSC.
*Z*	The objective function synthesized by *Z*_2_ and *Z*_3_, which is also called the comprehensive performance of LSSC.
Z*	The optimal value of *Z.*
*Z*_1_^min⁡^	The minimum of *Z*_1_ when not considering the objective functions *Z*_2_ and *Z*_3_.
*Z*′	The objective function synthesized by *Z*_1_, *Z*_2_, and *Z*_3_.
*Z* ^′∗^	The optimal value of *Z*′.
*β*	The delay coefficient of the order completion time permitted by its customer.
*ρ*	The coefficient of mass service effects obtained by LSI, which presents the cost reduction due to the increase of mass operations.
*w*	The customer order difference tolerance coefficient of LSI.
*θ*	The coefficient of the order differences, which reflects the degree of differences among each order in terms of customized degree in a certain order set. $\theta =\left(1/J_{0}\right)\overset{J_{0}}{\underset{j=1}{\sum }}\left(\Delta K_{j}/K_{j}\right)$
*i* = 1,2, 3,…, *I*_0_, *j* = 1,2, 3,…, *J*_0_	

Note: *k*, *T*_*i*_^ext^, and *T*_*ij*_^ext^ are decision variables.

**Table 2:** Basic data.

Parameter	*i*						
	*i* = 1	*i* = 2	*i* = 3	*i* = 4	*i* = 5	*i* = 6	*i* = 7
*T*_*i*_	10	9	10	15	8	12	8
*C*_*i*_	4	3	5	7	5	2	3
*C*_*i*_^ext^	6	5	7	9	7	5	5
*T*_*i*_^exp⁡^	13	8	7	13	10	8	12
*P*_*i*_	7	5	10	12	7	9	6
*D*_*i*_	4	3	8	10	5	7	5
*U*_*i*_	0.5	0.5	0.6	0.55	0.55	0.4	0.5
*T*_*i*+1_^+^	—	3	4	3	3.5	5	3.5
*T*_*i*+1_^−^	—	−3	−4	−5	−5	−4	−4

**Table 3:** Results of numerical example calculation.

Service stage	Service process	Normal service time		Additional service time	Actual service time
Mass stage	Process 1	10		0.0759	10.0759
	Process 2	9		0.2400	9.2400
	Process 3	10		−0.0929	9.9071

Customized stage	Process 4	Customer 1	12	1.4879	13.4879
		Customer 2	10	0.7492	10.7492
		Customer 3	11	−0.9107	10.0893
	Process 5	Customer 1	6	1.4837	7.4837
		Customer 2	7	0.0022	7.0022
		Customer 3	7.5	0.0113	7.5113
	Process 6	Customer 1	6	0.6990	6.6990
		Customer 2	7	0.0155	7.0155
		Customer 3	8	0.0102	8.0102
	Process 7	Customer 2	7	0.0000	7.0000
		Customer 3	10	−0.8458	9.1542

**Table 4:** The influence of β on comprehensive performance of LSSC.

*β*	*Z*
−0.09	0.8482
−0.08	0.8496
−0.05	0.8591
−0.02	0.8603
0	0.9055
0.02	0.9239
0.05	0.9324
0.08	0.9251
0.1	0.9178
0.12	0.9124
0.15	0.9085
0.18	0.8977

**Table 5:** The influence of *c* on scheduling result.

*c*	*Z*		Z*
	*k* = 4	*k* = 5	
0.02	*Z* = 0.9226	*Z* = 0.8439	*Z* = 0.9226
0.05	*Z* = 0.9297	*Z* = 0.8486	*Z* = 0.9297
0.10	*Z* = 0.9307	*Z* = 0.8511	*Z* = 0.9307
0.15	*Z* = 0.9310	*Z* = 0.8522	*Z* = 0.9310
0.2	*Z* = 0.9324	*Z* = 0.8530	*Z* = 0.9324
0.25	*Z* = 0.9324	*Z* = 0.8536	*Z* = 0.9324
0.3	*Z* = 0.9323	*Z* = 0.8536	*Z* = 0.9323
0.4	*Z* = 0.9324	*Z* = 0.8538	*Z* = 0.9324

**Table 6:** *Z* varied with *w*.

*w*	Optimal CODP	*Z*
0.05	No solution	No solution
0.06	*k* = 5	0.8530
0.1	*k* = 5	0.8530
0.15	*k* = 5	0.8530
0.2	*k* = 5	0.8530
0.25	*k* = 4	0.9324
0.3	*k* = 4	0.9324
0.35	*k* = 4	0.9324
0.4	*k* = 4	0.9324
0.45	*k* = 4	0.9324
0.5	*k* = 4	0.9324
0.55	*k* = 4	0.9324
0.6	*k* = 4	0.9324
0.65	*k* = 4	0.9324

**Table 7:** Results of *Z*′ varied with *ρ*.

*ρ*	*Z*′ when *k* = 4	*Z*′ when *k* = 5	Optimal CODP
0	0.7185	0.7034	*k* = 4
0.05	0.7250	0.7243	*k* = 4
0.10	0.7318	0.7558	*k* = 5
0.15	0.7417	0.7986	*k* = 5
0.2	0.7488	0.8637	*k* = 5

**Table 8:** The results of *Z*′ varied with *ρ* (*R* = 3).

*ρ*	*Z*′ when *k* = 4	*Z*′ when *k* = 5	Optimal CODP
0	0.6359	0.6268	*k* = 4
0.05	0.6461	0.6635	*k* = 5
0.10	0.6595	0.7098	*k* = 5
0.15	0.6734	0.7727	*k* = 5
0.2	0.6885	0.8732	*k* = 5

**Table 9:** The influence of *ρ* on the scheduling results when *R* changes.

*R*	*ρ*	*Z*′ when *k* = 4, (*R* = 3)	*Z*′ when *k* = 5, (*R* = 3)	Optimal CODP	The threshold of *ρ* when the optimal CODP changes from *k* = 4 to *k* = 5
3	0	0.6359	0.6268	*k* = 4	0.017
	0.05	0.6461	0.6635	*k* = 5	
	0.10	0.6595	0.7098	*k* = 5	
	0.15	0.6734	0.7727	*k* = 5	
	0.2	0.6885	0.8732	*k* = 5	

2	0	0.6625	0.6530	*k* = 4	0.022
	0.05	0.6722	0.6838	*k* = 5	
	0.10	0.6836	0.7247	*k* = 5	
	0.15	0.6961	0.7814	*k* = 5	
	0.2	0.7090	0.8684	*k* = 5	

1	0	0.7185	0.7034	*k* = 4	0.05
	0.05	0.7250	0.7243	*k* = 4	
	0.10	0.7318	0.7558	*k* = 5	
	0.15	0.7417	0.7986	*k* = 5	
	0.2	0.7488	0.8637	*k* = 5	

1/2	0	0.7738	0.7526	*k* = 4	0.082
	0.05	0.7793	0.7685	*k* = 4	
	0.10	0.7836	0.7894	*k* = 5	
	0.15	0.7900	0.8179	*k* = 5	
	0.2	0.7960	0.8597	*k* = 5	

1/3	0	0.8039	0.7787	*k* = 4	0.115
	0.05	0.8058	0.7890	*k* = 4	
	0.10	0.8093	0.8052	*k* = 4	
	0.15	0.8149	0.8247	*k* = 5	
	0.2	0.8167	0.8588	*k* = 5	
